# Anti-VEGF immunotherapy with HEBERSaVax suppresses melanoma growth and metastasis via angiogenesis blockade and enhanced T-cell infiltration

**DOI:** 10.3389/fimmu.2025.1667651

**Published:** 2025-12-19

**Authors:** Yanelys Morera-Díaz, Josune García-Sanmartín, Camila Canaán-Haden, Mónica Bequet-Romero, Isabel Gonzalez-Moya, Marta Ayala-Ávila, Srdan Tadic, Pablo Garrido, Judit Narro-Íñiguez, Dasha Fuentes-Morales, Johanna Bernáldez-Sarabia, Blanca J. Valdovinos-Navarro, Alexei F. Licea-Navarro, Alfredo Martínez

**Affiliations:** 1 Cancer Immunotherapy Group, Direction of Biomedical Research, Center for Genetic Engineering and Biotechnology (CIGB), BioCubaFarma, Havana, Cuba; 2 Angiogenesis Unit, Oncology Area, Center for Biomedical Research of La Rioja (CIBIR), Logroño, Spain; 3 Experimental Biomodels Group, Direction of Gnotobiotic Animals, National Center for the Production of Laboratory Animals (CENPALAB), BioCubaFarma, Havana, Cuba; 4 Molecular Immunology and Biotoxins Laboratory, Biomedical Innovation Department, Centro de Investigación Científica y de Educación Superior de Ensenada (CICESE), Ensenada, Mexico

**Keywords:** anti-angiogenic therapy, cancer immunotherapy, HEBERSaVax, melanoma treatment, tumor microenvironment

## Abstract

**Introduction:**

Targeting tumor angiogenesis through vascular endothelial growth factor (VEGF) blockade represents a promising strategy for melanoma treatment. Here, we evaluate the therapeutic potential of HEBERSaVax, an anti-VEGF active immunotherapy, in aggressive B16-F10 syngeneic melanoma models.

**Methods:**

The antitumor activity of HEBERSaVax, formulated with aluminum phosphate adjuvant, was evaluated in C57BL/6 mice using two distinct B16-F10 melanoma models (i): subcutaneous inoculation to assess primary tumor growth inhibition, and (ii) intravenous inoculation to quantify lung metastasis suppression. Tumor vasculature and microenvironment changes were analyzed via immunohistochemistry (CD31, α-SMA, CD4, CD8).

**Results:**

HEBERSaVax significantly reduced primary tumor volume and weight in subcutaneous implants compared to adjuvant controls. Histopathological analysis revealed potent angiogenesis inhibition, decreased CD31^+^ vessel density, and vascular remodeling. Concomitant with tumor control, we observed changes in the tumor microenvironment, including a reduction in α-SMA^+^ pericytes and an increase in the infiltration of CD4^+^ and CD8^+^ T cells. In the metastatic model, HEBERSaVax-treated mice showed fewer pulmonary nodules versus controls.

**Discussion:**

Our results demonstrate that HEBERSaVax mediates dual antitumor efficacy by simultaneously suppressing VEGF-dependent angiogenesis and promoting immune-related changes in the melanoma microenvironment. These findings support the further development of HEBERSaVax as a promising active immunotherapy for VEGF-driven advanced melanoma.

## 1 Introduction

HEBERSaVax (also known as CIGB-247 in earlier preclinical publications) represents a novel active immunotherapy platform targeting vascular endothelial growth factor (VEGF), a pivotal tumor angiogenesis and immune suppression regulator. The vaccine formulation incorporates recombinant human VEGF_121_ produced in bacterial fermentation systems, engineered with three critical amino acid substitutions to abrogate receptor binding and angiogenic activity (known as CIGB-247 antigen) (1). This innovation builds upon extensive preclinical optimization, where aluminum phosphate (AP) adjuvant emerged as superior to aluminum hydroxide (AlOH) or other adjuvants (e.g., VSSP, CAF01), due to its enhanced antigen stability, immune tolerance breaking, and induction of high-affinity anti-VEGF antibodies with robust antitumor efficacy in mammary cancer models (2). This optimization enabled HEBERSaVax’s dual mechanism simultaneous VEGF neutralization and adaptive immunity induction - while maintaining clinical-grade stability. The clinical translation of HEBERSaVax has demonstrated a favorable safety profile and consistent immunogenicity in phase 1/2 trials for various solid tumors (3–5). Subsequent phase 2 investigations have shown promising preliminary efficacy in ovarian cancer (6) and hepatocellular carcinoma. However, despite melanoma’s well-characterized dependence on VEGF signaling, the potential of HEBERSaVax in this malignancy remains unexplored.

Melanoma, a highly aggressive and metastatic skin cancer, represents an ideal candidate for HEBERSaVax therapy. VEGF plays a pivotal role in melanoma progression by promoting angiogenesis, immunosuppression, and metastasis, underscoring its relevance as a therapeutic target for metastasis prevention and/or reduction (7, 8). Current anti-angiogenic strategies, including VEGF-A-neutralizing monoclonal antibodies (e.g., bevacizumab), VEGFR-targeted tyrosine kinase inhibitors (e.g., sorafenib, axitinib), and VEGF decoy receptors (e.g., aflibercept), have shown limited clinical efficacy as monotherapies (9, 10), although they work well in combination with other drugs. Challenges such as rapid development of resistance, dose-limiting toxicities, and chronic administration highlight the urgent need for more sustainable approaches.

The selection of VEGF as the immunogen for HEBERSaVax is underpinned by its well-established dual role in melanoma progression. As a stable, stromal target ubiquitously overexpressed in melanoma, VEGF drives both angiogenesis and fosters an immunosuppressive tumor microenvironment (TME) (7, 11). Its actions include inhibiting dendritic cell maturation, recruiting myeloid-derived suppressor cells (MDSCs) and regulatory T cells (Tregs), and impairing T-cell infiltration and function, thereby creating a physical and functional barrier to antitumor immunity (11, 12). This rationale is strongly supported by clinical and preclinical evidence. Although monotherapy with other anti-VEGF agents (e.g., bevacizumab) has shown limited efficacy in melanoma, it consistently demonstrates the ability to remodel the TME, normalize tumor vasculature, and enhance T-cell infiltration (13), providing a compelling basis for its use in combination strategies. Therefore, targeting VEGF with an active immunotherapy like HEBERSaVax is a strategic approach to simultaneously disrupt tumor vascularization and reverse immune suppression, potentially overcoming limitations of passive antibody therapies and creating a more favorable environment for antitumor immunity.

HEBERSaVax represents a promising therapeutic advance by inducing durable, active anti-VEGF immunity with a favorable safety profile (1). However, three critical limitations must be addressed to optimize its clinical potential in melanoma. First, while early preclinical studies with aluminum hydroxide (AlOH)-adjuvanted formulations demonstrated safety, they showed inadequate antitumor efficacy (14), highlighting the need for alternative adjuvants. Second, the immunological impact of the current (AP)-formulated vaccine on the TME remains uncharacterized. Third, despite VEGF’s well-established role in metastatic progression through angiogenesis, immune evasion, and pre-metastatic niche formation (12–14), no preclinical studies have evaluated HEBERSaVax in metastatic melanoma models, creating a significant gap in understanding its potential to control disseminated disease. Addressing these knowledge gaps will be essential for positioning HEBERSaVax as a viable therapeutic option across the melanoma disease spectrum.

Here, we evaluate AP-formulated HEBERSaVax in the immunocompetent B16-F10 melanoma model (15), a gold standard for studying VEGF-driven angiogenesis and metastasis. For the first time, we demonstrate its dual mechanism of action: blocking angiogenesis (reducing CD31^+^ microvessels and α-SMA^+^ pericytes) while remodeling the TME through increased CD4^+^/CD8^+^ T-cell infiltration. These findings highlight its potential to target both primary and metastatic disease. By bridging critical gaps between prior research and clinical translation, this study provides a preclinical rationale for developing HEBERSaVax as a combinatorial immunotherapy for advanced melanoma.

## 2 Materials and methods

All chemicals, materials, and their providers are listed in **Supplementary Table S1**.

### 2.1 Cell lines

Mouse melanoma cell line B16-F10 was obtained from the American Tissue Culture Collection (ATCC-CRL-6475, Manassas, VA, USA) and maintained in RPMI supplemented with 10% FBS at 37°C, in an atmosphere containing 5% CO2.

### 2.2 Animals and ethical approval

For the subcutaneous B16-F10 model, sixteen 8-week-old C57BL/6 mice (8 males and 8 females) were purchased from a commercial breeder (Inotiv, West Lafayette, IN, USA). Mice were randomly distributed in groups of four and housed at the CIBIR animal facility under constant temperature (21 ± 2°C) and humidity on a 12-hour light/dark cycle, with lights on at 7:00 am. Standard food and sterile water were available ad libitum. All procedures involving animals were carried out following the European Communities Council Directive (2010/63/EU) and Spanish legislation (RD53/2013) on animal experiments and with approval from the ethical committee on animal welfare of our institution (Órgano Encargado del Bienestar Animal del Centro de Investigación Biomédica de La Rioja, OE-BA-CIBIR, procedure number AMR17, approved on November 30th, 2020).

For the B16-F10 pulmonary metastasis model, thirty 8-week-old female C57BL/6 mice were purchased and maintained at the National Center for Animal Breeding (CENPALAB, Havana, Cuba) animal facility following the Cuban guidelines for the care and use of laboratory animals. Animal studies were conducted according to a protocol approved by the Institutional Animal Care and Use Committee of the National Center for Laboratory Animal Breeding (CENPALAB) (permit number 17/17).

### 2.3 Vaccine antigen and adjuvants

The antigen used in this study is a recombinant fusion protein, representative of human VEGF_121_ isoform, that shares an 87% homology with murine VEGF_120_. The human growth factor fully activates the murine VEGFRs; nevertheless, the vaccine antigen has been mutated into the VEGFR2 binding site (Arg82, Lys84, and His86 →Glu) to avoid unintended angiogenesis (14). The lyophilized antigen was produced in vials of 400 μg by the Development Unit of the Center of Genetic Engineering and Biotechnology (CIGB, Havana, Cuba) (lot 2121VE8/0). The desired antigen amount was mixed with AP at the moment of vaccination, and formulated with 0.70 mg of Al^3+^ per dose. At the moment of vaccination, antigen vials were dissolved in pre-calculated amounts of injection water up to a final volume never exceeding 0.25 mL per mouse and injection dose.

### 2.4 Subcutaneous tumor model

Mice were randomly divided into two groups of eight animals each. They received four subcutaneous injections of either (a) vehicle combined with AP adjuvant preparations (control animals) or (b) 200 µg of the CIGB-247 antigen mixed with AP (0.70 mg Al^3+^) (HEBERSaVax) on a biweekly schedule. This scheme was selected based on previous preclinical data, later validated in the clinical context (2, 4).

Six days after each immunization, every mouse was bled to determine the titers of anti-VEGF antibodies. Three days after the second immunization, a suspension of B16-F10 cells (5 × 10^4^ cells per mouse) in 0.1 mL RPMI was subcutaneously injected into the left rear flank of each mouse. Tumor growth was measured with calipers, following the formula (Length × Width^2^)/2, thrice a week for 3 weeks. On day 56, the animals were sacrificed by an intraperitoneal injection of 200 mg/Kg sodium pentobarbital (Dolethal, Vetoquinol, Madrid, Spain), and blood and tumors were collected. Small tumor pieces were fixed in 10% neutral buffered formalin, dehydrated, paraffin-embedded, and sectioned for immunohistochemical analysis.

### 2.5 ELISA for anti-VEGF antibody titers

Sera samples were analyzed for anti-human VEGF by ELISA. EIA 96-well plates were coated overnight at 4°C with 10 µg/mL of GSThVEGF_121_ in PBS. After three washes with 0.1% Tween 20 in PBS, the plates were blocked with 2% skim milk in PBS for 1 h at 22°C, followed by new washes. PBS-diluted sera were added to wells and incubated for 1 h at 22°C. Wells were washed three times and incubated with goat anti-mouse IgG HRP-conjugated antibodies (Sigma). After incubation for 1 h at 22°C, plates were re-washed and incubated with substrate chromogen solution (OPD 0.75 mg/mL, hydrogen peroxide 0.015%, in citrate-phosphate buffer, pH 5.5) for 15 min. The reaction was stopped by adding 50 µL of 2 M sulfuric acid solution, and the absorbance was read at 492 nm in a BioRad microtiter plate reader. The 492 nm absorbance value corresponding to a PBS sample was subtracted from all the obtained diluted serum or plasma values.

Non-linear regression curves were adjusted for the OD values obtained from the dilutions of each sample, and the value corresponding to 3 standard deviations greater than the mean OD obtained in wells containing non-immune samples was interpolated and considered the titer.

### 2.6 ELISA for inhibition of VEGF/KDR binding

Vaccine-induced serum neutralizing capacity was assessed via competition ELISA. Plates were coated overnight at 4°C with 10 µg/mL of GSThVEGF_121_ in PBS. After three washes with 0.1% Tween 20 in PBS, the plates were blocked with 2% skim milk in PBS for 1 h at 22°C, followed by new washes. Serial dilutions of sera or different concentrations of purified serum antibodies were added and incubated for 1 h at 22°C. Then, 125 µg of recombinant human VEGF receptor 2/Fc chimera (KDR-Fc; Sigma) were added to the wells and incubated for 45 min at 22°C. Wells were washed three times and incubated with goat anti-human Fc HRP-conjugated antibodies. After washing, plates were incubated with substrate–chromogen solution (OPD 0.75 mg/mL, hydrogen peroxide 0.015%, in citrate phosphate buffer, pH 5.5) for 15 min. Reaction was stopped by adding 2 M sulphuric acid, and the absorbance was read at 492 nm in a BioRad microtiter plate reader.

The ability of sera to neutralize VEGF–VEGFR2 interaction was expressed as a per-cent of the maximum binding of KDR-Fc to GST-hVEGF_121_ in the presence of pre-immune serum, by the formula:


$$\begin{array}{c} \%\;Inhibition\;of\;VEGF/KDRFc\;interaction\\ \;=100–(A492\;nm\;immune\;serum/A492\;nm\;preimmune\;serum)\times 100. \end{array}$$

### 2.7 *In vitro* cytotoxicity assays

*In vitro* cytotoxicity assays were performed using spleen cells isolated from immunized mice without tumor challenge. Twelve mice were allocated into two groups (n=6/group) and received four biweekly subcutaneous injections of either: (a) vehicle with AP adjuvant (control group), or (b) 200 µg CIGB-247 antigen combined with AP (HEBERSaVax group). Seven days post-final immunization, spleen cells were aseptically harvested. Freshly isolated splenocytes were co-cultured at a 50:1 effector-to-target ratio with CFSE-labeled B16-F10 cells. After 24 hours of co-culture, viable (CFSEhi) tumor cells were quantified. Specific cytotoxicity was calculated by comparing results to the adjuvant-only control group, with data expressed as the number of CFSE-positive B16-F10 cells remaining.

### 2.8 Experimental metastasis protocol

The effect of immunization on spontaneous metastasis formation was assessed using the B16-F10 experimental lung metastasis model. Mice were randomly divided into two groups of fifteen animals each and received four bi-weekly subcutaneous injections as described in 2.4. Twelve days after the second immunization, all mice were intravenously injected with 5 × 10^5^ B16-F10 tumor cells in 0.1 mL of RPMI via the lateral tail vein. Twenty-two days post-tumor challenge, all mice were anesthetized through intraperitoneal administration of a combination of Ketamine/Diazepam/Atropina (50/5/1 mg/Kg) (AICA, Havana, Cuba), followed by euthanasia via cervical dislocation. Lungs were examined for macroscopic metastases using a dissecting microscope immediately after excision or fixation in Bouin’s solution. Lung weight was also measured as an indicator of metastatic burden.

### 2.9 Immunohistochemistry

Tissue sections (3 μm-thick) were dewaxed in xylene, and endogenous peroxidase was blocked with 3% H_2_O_2_ in methanol for 15 min. Samples were rehydrated and subjected to antigen retrieval (10 mM sodium citrate, 0.5% Tween 20, pH 6.0, 20 min at 95°C). Nonspecific binding was blocked for 30 min by exposure to 5% normal donkey serum (Jackson ImmunoResearch Laboratories, West Grove, PA, USA) or 2.5% normal horse serum (Vector Laboratories, Newark, CA, USA).

Since the anti-α-SMA antibody was generated in mice, potential mouse IgG binding sites were blocked by overnight incubation with a 1:100 dilution of F(ab) fragment donkey anti-mouse IgG (Jackson ImmunoResearch Laboratories) at 4°C.

All tissue sections were incubated with primary antibodies (**Supplementary Table S2**) overnight at 4°C. The next day, after washing, a specific polymer containing horseradish peroxidase was used to detect the antibodies (**Supplementary Table S3**). After several washes, peroxidase activity was detected with the Bajoran Purple (BJP) chromogen kit (catalog number BJP811, Biocare Medical, Pacheco, CA, USA) to avoid confusion with the brown color of intrinsic melanin. Slides were lightly counterstained with hematoxylin and observed and recorded with a Leica DM 6000 B microscope equipped with a digital camera (Leica, L´Hospitalet de Llobregat, Barcelona, Spain).

### 2.10 Quantification of immunohistochemical signals

Ten random images per animal were captured and analyzed using ImageJ^®^ free software (NIH, Bethesda, Maryland, USA). The area to be analyzed for each image was selected and separated from the rest of the field using the image brush tool. Using the color deconvolution plugin, the analyzed field automatically measured the BJP-positive and hematoxylin areas (the whole nuclear area). Each image was transformed into an 8-bit file and then processed into a binary color image. For α-SMA expression, a score was calculated by dividing the positively stained area by the whole selected area.

For CD31, the number of vessels per area was calculated by counting the number of BJP-positive vessels over the selected region. The original BJP image was deconvoluted, separating into hematoxylin staining for the nuclei and BJP-staining for CD31 protein expression. The BJP-staining was later transformed to an inverted 8-bit gray-level digital image and applied a specific threshold. The pixels of this image were dilated. This dilated image was skeletonized, and the number of vessels was counted from 100 pixel^2^ to Infinity (**Supplementary Figure S1**).

Ki67, FOXP3, CD4, and CD8 presence was calculated by dividing the number of BJP-positive cells by the total number of hematoxylin-positive nuclei.

### 2.11 Statistical analysis

Direct or transformed (Log10) data that passed the normality test (Kolmogorov–Smirnov normality test) and showed variance homogeneity (Bartlett’s test) were analyzed with parametric tests. Statistical analyses between the two groups were performed using Student’s t-test. In contrast, comparisons of multiple groups were analyzed using one-way or two-way ANOVA, followed by Sidak´s multiple comparison analysis. Even after transformations, data that did not fulfill normality and/or variance homogeneity tests were analyzed using nonparametric tests such as Kruskal-Wallis or Mann-Whitney tests. GraphPad Prism 10 for Windows version (GraphPad Software, San Diego, CA, USA) was used for calculations. Statistical significance is indicated as follows: *, P < 0.05; **, P < 0.01; ***, P < 0.001; ****, P < 0.0001; ns = not significant. P values < 0.05 were considered statistically significant.

## 3 Results

### 3.1 Anti-tumor efficacy of HEBERSaVax correlates with anti-VEGF antibody titers in subcutaneous melanoma-bearing mice

**Figure 1:**
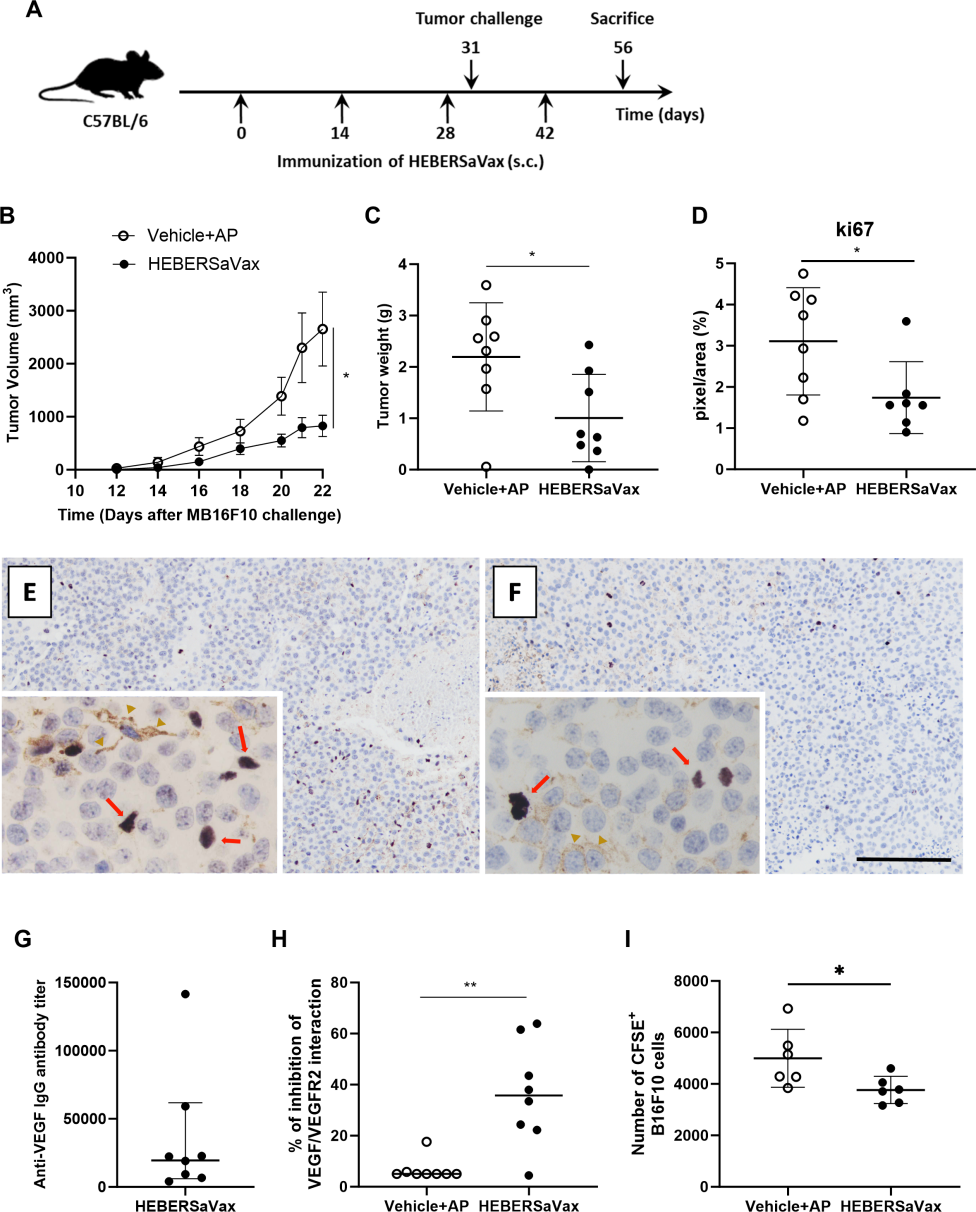
HEBERSaVax immunization inhibits B16-F10 melanoma growth and induces anti-VEGF immunity. **(A)** Experimental design: Mice (n=7-8/group) received biweekly immunizations with HEBERSaVax (CIGB-247 antigen+AP) or vehicle + AP (Vehicle+AP). Three days after the third dose, animals were challenged subcutaneously with 2×10^5^ B16-F10 cells and received one additional immunization during tumor progression. **(B)** Tumor growth kinetics (mean ± SEM). **(C)** Endpoint tumor weights. **(D)** Quantification of Ki67^+^ nuclei (10 fields/sample). **(E, F)** Representative tumor sections stained for Ki67 (proliferation marker) from control **(E)** and HEBERSaVax-treated **(F)** mice (scale bar = 200 µm; 50 µm for insets). Red arrows point to Ki67^+^ tumor cells while brown arrowheads point to melanin pigment. **(G)** Anti-human VEGF IgG titers in individual mice. **(H)** Serum-mediated inhibition of VEGF/KDR binding (percentage inhibition, duplicate measurements). **(I)** Direct tumor cell cytotoxicity: Splenocytes from HEBERSaVax-immunized mice (n=6) versus vehicle controls (n=6) were co-cultured with syngeneic B16-F10 cells at a 50:1 effector: target ratio. Each symbol represents an individual animal’s mean cytotoxicity from technical replicates **(G)**. Bars show group means ± SD **(C, D, G, I)** or median **(H)**. Statistical analysis by unpaired t-test (*p<0.05, **p<0.01). Significance was assessed by two-way ANOVA **(B)**, unpaired t-test (C, D, G, I), or Mann-Whitney test **(H)** (*p<0.05, **p<0.01) representative of two independent experiments.

To evaluate the antitumoral effect on B16-F10 melanoma, C57BL/6 mice were immunized with 200 μg of either antigen or vehicle and challenged with subcutaneous tumor cells. On day 56 post-challenge (14 days after the last immunization), tumors were surgically excised and weighed (**Figure 1A**). HEBERSaVax administration significantly reduced tumor volume compared to the negative control (**Figure 1B**, two-way ANOVA, significant interaction F = 4.460, mean difference: 673.1 mm^3^, 95% CI [-7.206 to 1353], p<0.05, Sidak´s multiple comparison test). Consistent with tumor volume measurements, HEBERSaVax-treated mice exhibited a significant 45.74% reduction in post-mortem tumor weight compared to the vehicle-adjuvant group (**Figure 1C**, mean difference: -1.191 ± 0.4784 g, 95% CI [-2.217 to -0.1645], p=0.0260, unpaired t-test).

Immunohistochemical analysis of Ki67 expression in B16-F10 tumor sections revealed significant anti-proliferative effects following HEBERSaVax treatment. Control tumors exhibited high proliferative activity as shown by abundant Ki67^+^ cells (**Figure 1E**). In contrast, vaccinated animals (**Figure 1F**) showed a marked 44% reduction in Ki67^+^ cells compared to vehicle-treated controls (**Figure 1D**, mean difference: -0.5448 ± 0.2636%, 95% CI [-1.114 to 0.02463], p=0.0296, unpaired t-test), demonstrating the treatment’s potent growth-inhibitory effects.

We evaluated the VEGF-specific antibody response to characterize the humoral immune response induced by immunization with HEBERSaVax. Serial dilutions from 1:500 of the individual sera from mice immunized with vehicle and adjuvant produced an absorbance value similar to the blanks used in the experiment. HEBERSaVax-immunized mice developed anti-human VEGF IgG antibodies, with titers ranging from 1:4.000 to 1:140.000 one week after the fourth dose (**Figure 1G**). Higher antibody titers correlated with reduced tumor volume (r = -0.6595, p = 0.0486, Pearson test), suggesting an association between humoral response and anti-tumor efficacy.

A competitive ELISA assay was also performed to test the ability of the antibodies induced by HEBERSaVax to hamper the interaction between VEGF and its central cognate receptor, KDR. HEBERSaVax immune sera efficiently blocked VEGF/KDR interaction, producing an 86% increase compared to sera from negative control animals (**Figure 1H**, mean difference: 30.74%, 95% CI [17.20 to 55.75], p=0.0071, Mann–Whitney test). Complementary studies in the same mouse strain demonstrated VEGF-specific cellular responses capable of efficiently lysing B16-F10 melanoma cells *ex vivo* showing a marked 75% reduction (**Figure 1I**, mean difference: -1230 ± 508.6%, 95% CI [-2363 to -96.81], p=0.0362, unpaired t-test).

As a surrogate of good health, the weight of all animals was recorded biweekly, and there were no significant differences between the experimental groups, indicating that the vaccine did not elicit serious adverse effects (**Supplementary Figure S2**). Despite the increase in direct cytotoxicity, no signs of vitiligo were observed.

### 3.2 HEBERSaVax exhibits potent anti-angiogenic effects and coordinated immune-vascular modulation

**Figure 2:**
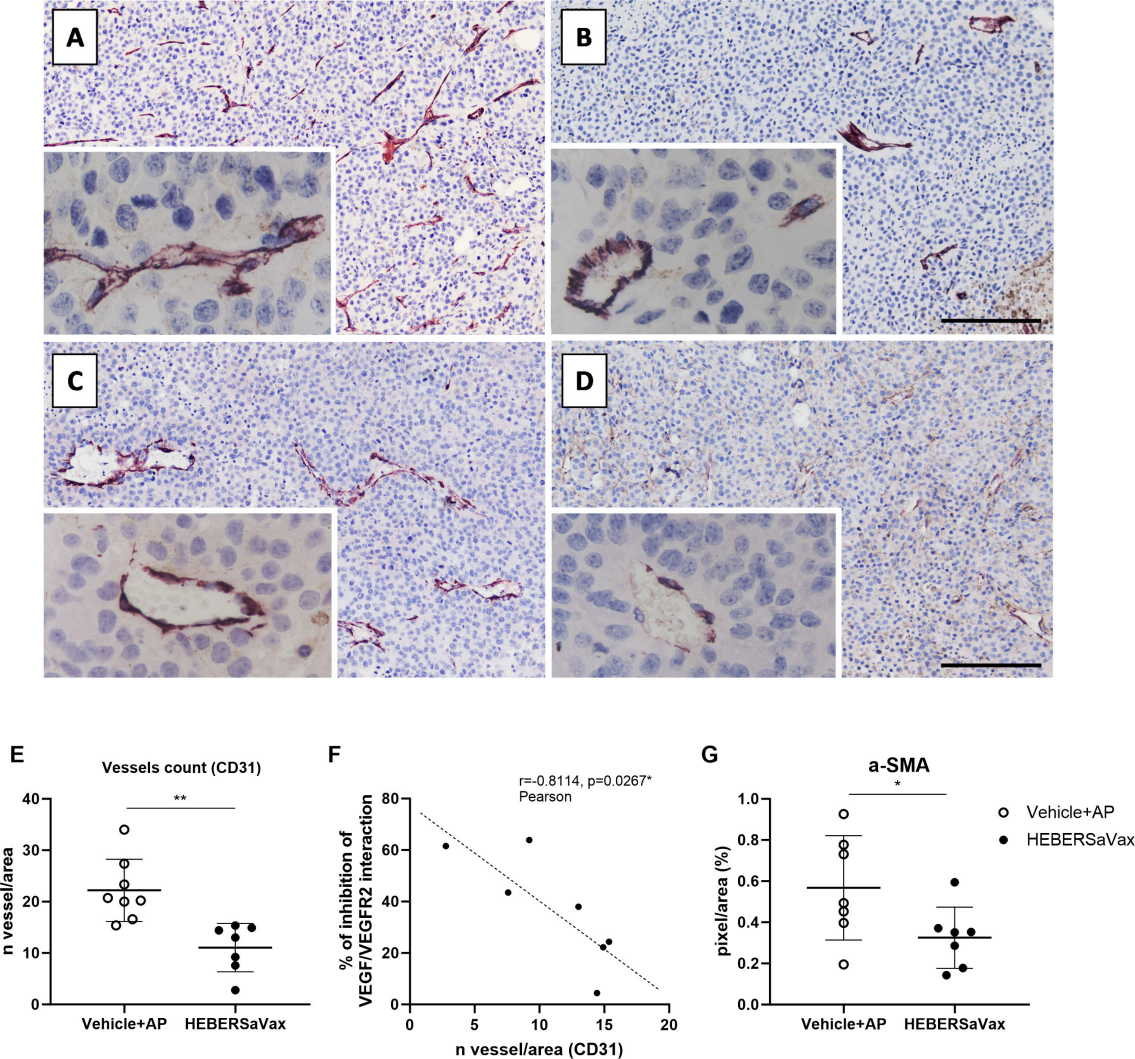
HEBERSaVax treatment modulates tumor angiogenesis in B16-F10 melanoma. Representative immunohistochemical images of tumor sections from vehicle-treated **(A)** and HEBERSaVax-treated **(B)** mice stained for CD31 (endothelial marker; scale bar = 200 µm; 50 µm for insets). Representative αSMA immunostaining of **(C)** control and **(D)** treated tumors (pericyte/vascular smooth muscle marker; scale bar = 200 µm; 50 µm for insets). Quantification of CD31^+^ microvessel density (vessels/mm²) **(E)**. **(F)** Inverse correlation between percentage inhibition, VEGF/VEGFR2 interaction, and CD31^+^ microvessel density (vessels/mm²). **(G)** Quantification of αSMA+ immunoreactive area. All angiogenesis-related quantifications represent mean ± SD. Data points represent individual mice (n=7-8/group; 10 fields analyzed per mouse). Statistical analyses: Angiogenesis and immune cell infiltration markers were analyzed by unpaired t-test **(E, G)**, and correlation analyses used the Pearson test **(F)** (*p<0.05; **p<0.01).

To assess anti-angiogenic effects, B16-F10 tumors were analyzed by CD31/αSMA immunohistochemistry experiments (**Figures 2A-D**). HEBERSaVax treatment significantly reduced vascular density (**Figure 2B**), demonstrating a 51% decrease in CD31^+^ microvessels compared to vehicle-AP controls (**Figure 2A**) (**Figure 2E**; mean difference: -11.17 ± 2.832 vessels/mm², 95% CI [-17.28 to -5.049], p=0.0017, unpaired t-test). This reduction strongly correlated with VEGF/VEGFR blockade (**Figure 2F**; r=−0.8114, p=0.0267, Pearson test), suggesting a mechanistic link.

Vessel maturation analysis revealed a 42.7% reduction in αSMA^+^ pericytes in treated tumors (**Figure 2D**) versus controls (**Figure 2C**) (**Figure 2G**; mean difference: -0.2423 ± 0.1111%, 95% CI [-0.4844 to -0.0002229], p=0.0249, unpaired t-test). Together, these findings demonstrate HEBERSaVax’s potent disruption of tumor vasculature, as evidenced by significant reductions in endothelial and pericyte markers.

**Figure 3:**
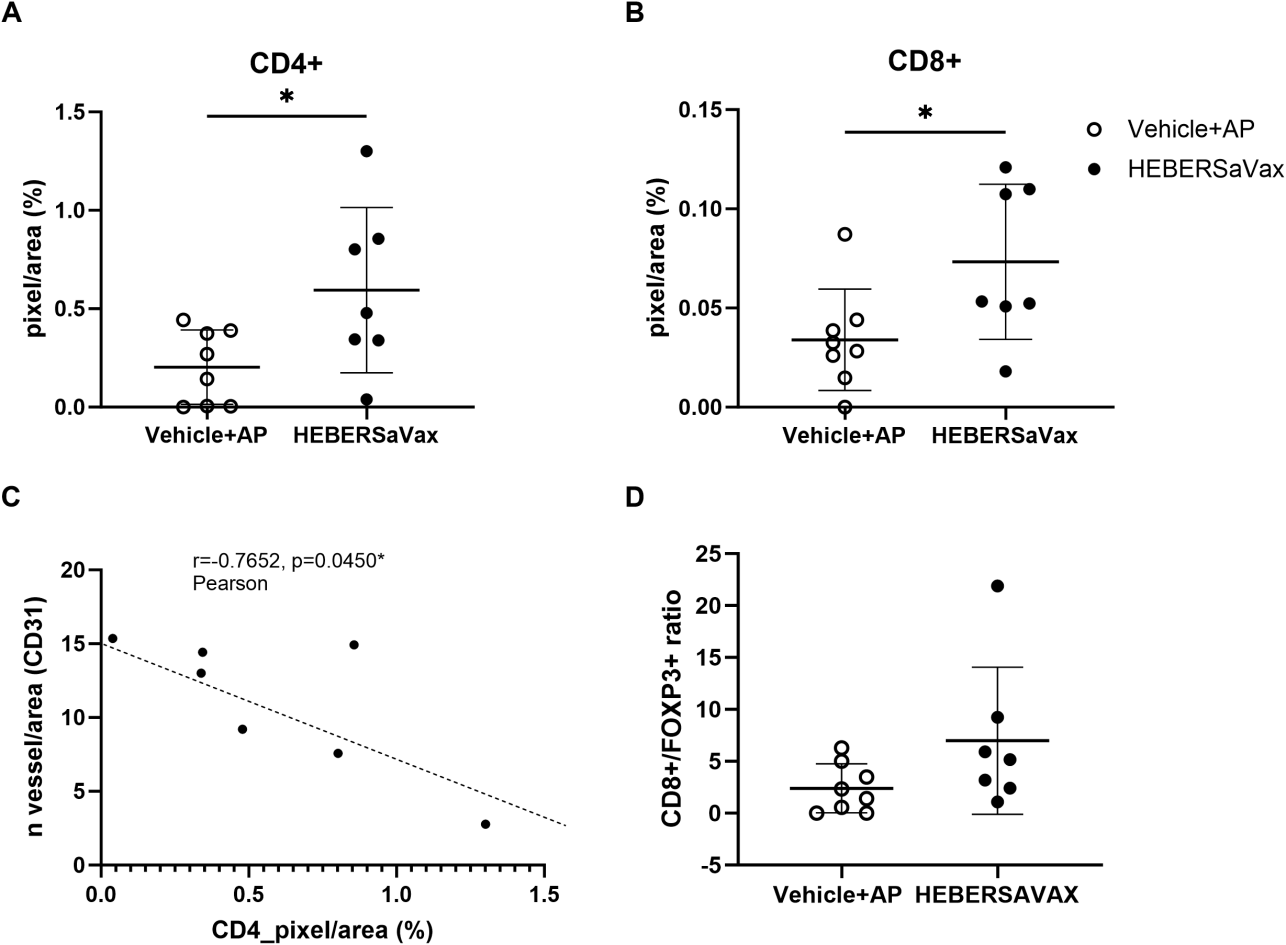
HEBERSaVax treatment modulates immune infiltration in B16-F10 melanoma. Quantification of tumor-infiltrating **(A)** CD4^+^ and **(B)** CD8^+^ T lymphocytes, expressed as percentage of immunoreactive cells. **(C)** Inverse correlation between CD31^+^ microvessel density (vessels/mm²) and CD4^+^ T cell infiltration (pixels/mm²). **(D)** Immunohistochemical analysis of CD8^+^/FOXP3^+^ cell ratio in B16-F10 tumor. Data points represent individual mice (n=7-8/group; 10 fields analyzed per mouse). Bars indicate mean ± SD. Statistical analyses: Angiogenesis and immune cell infiltration markers were analyzed by unpaired t-test **(A, B, D)**, and correlation analyses used Pearson test **(C)** (*p<0.05).

Immunohistochemical analysis revealed that HEBERSaVax treatment significantly increased infiltration of both CD4^+^ T cells by 65.74% (**Figure 3A**; mean difference: 0.3907 ± 0.1643%, 95% CI [0.03574 to 0.7456], p=0.0334) and increased infiltration of CD8^+^ T cells by 53.64% (**Figure 3B**; mean difference: 0.03931 ± 0.01683%, 95% CI [0.002942 to 0.07568], p=0.0362) compared to controls. This immunomodulatory effect is consistent with established consequences of VEGF pathway inhibition. Furthermore, the strong inverse correlation between CD4^+^ T cell density and CD31^+^ microvessel density (**Figure 3C**; r=−0.7652, p=0.0450) suggests a potential link between vascular remodeling and improved immune cell access.

Analysis of Treg infiltration revealed an apparently higher CD8^+^/FOXP3^+^ ratio in HEBERSaVax-treated mice (6.984 vs. 2.391 in controls), although this difference did not reach statistical significance (**Figure 3D**; p=0.1066, unpaired t-test). Probably, the limited number of FOXP3^+^ cells (average <5% of CD4^+^ population) and the known low Treg infiltration characteristic of the B16-F10 model constrained statistical power. While sample availability precluded comprehensive exhaustion marker analysis, these preliminary findings provide valuable pilot data for future mechanistic studies with optimized experimental designs.

### 3.3 HeberSAVax reduces the number of experimental lung metastases produced by B16-F10 tumor cells

**Figure 4:**
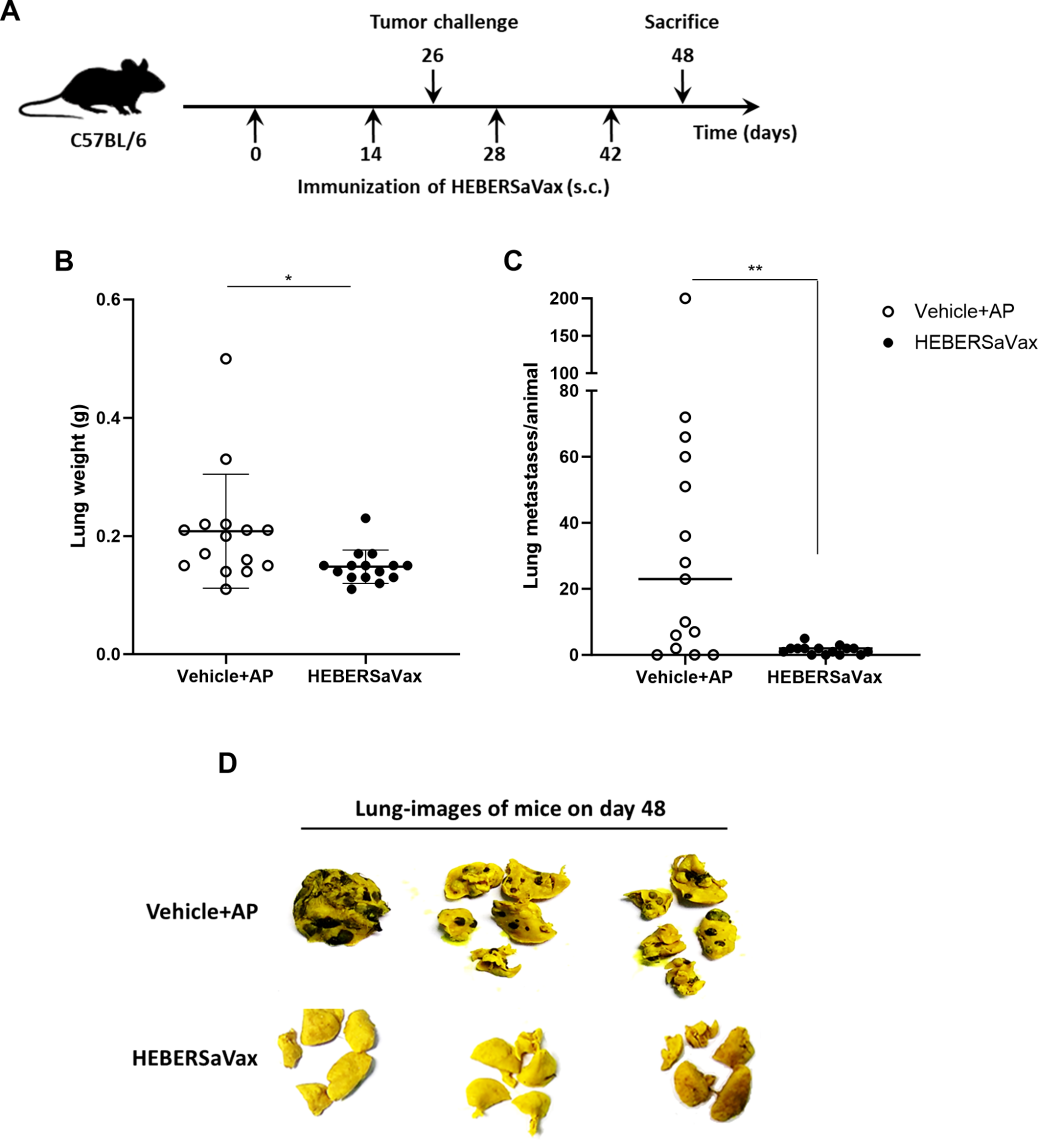
HEBERSaVax immunization significantly inhibits B16-F10 lung metastasis in C57BL/6 mice. **(A)** Experimental timeline: mice (n=15/group) received biweekly immunizations with either HEBERSaVax or vehicle control. Twelve days after the second immunization, animals were challenged via tail vein injection with 5×10^5^ B16-F10 cells and euthanized on day 48. **(B)** Metastatic burden assessed by lung weight. **(C)** Quantification of the number of metastatic foci by stereomicroscopy. **(D)** Representative photographs of lungs fixed in Bouin´s solution. Data points represent individual animals; bars indicate **(B)** mean ± SD or **(C)** median. Statistical significance was determined by unpaired t-test **(B)** or Mann-Whitney test **(C)** (*p<0.05, **p<0.01).

To evaluate the anti-metastatic efficacy of HEBERSaVax, we established a B16-F10 experimental lung metastasis model in C57BL/6 mice by intravenous administration of tumor cells following the second immunization (**Figure 4A**). At day 22 post-challenge, lungs from HEBERSaVax-treated mice exhibited significantly lower whole-lung weights compared to adjuvant-vehicle-treated controls (**Figure 4B**; mean difference: -0.2813 ± 0.1062 g, 95% CI [-0.4990 to -0.06370], p=0.0132, unpaired t-test), indicating reduced metastatic burden. Quantitative analysis demonstrated a significant reduction in metastatic foci count in vaccinated mice (**Figure 4C**; median difference: -21 nodules/lung, p=0.0032; Mann-Whitney test). Macroscopic examination revealed extensive black B16-F10 colonies on the lung surfaces of control animals. In contrast, HEBERSaVax-treated lungs displayed substantially fewer metastatic lesions (**Figure 4D**). Together, these findings provide compelling evidence for HEBERSaVax’s potent anti-metastatic activity against B16-F10 lung colonization.

## 4 Discussion

Results presented herein demonstrate that HEBERSaVax exerts dual anti-tumor activity in aggressive B16-F10 melanoma by simultaneously targeting both vascular and immune components. The vaccine significantly reduced tumor growth, disrupted tumor vascularization, enhanced T-cell infiltration, and suppressed metastatic dissemination, thus highlighting its potential as a multifaceted therapeutic strategy.

While phase 1–2 trials established HEBERSaVax’s safety profile in multiple solid tumors (1, 6), its impact on melanoma, a disease with distinct VEGF signaling and immune evasion, remained uncharacterized. VEGF is a compelling target in melanoma, where it orchestrates both tumor progression through angiogenesis and immune suppression via T-cell exclusion and MDSC recruitment. Clinical evidence supports this, showing VEGFR expression in most metastatic melanomas (21) and a functional dependence on VEGFR-2 signaling in many cell lines (7). Indeed, VEGF can function as an autocrine growth factor for such VEGFR-positive human tumors (20).

The B16-F10 syngeneic model was selected for this study due to its well-established VEGF-driven angiogenesis, reproducible metastatic pattern, and immunocompetent microenvironment, which collectively provide a stringent system for evaluating the dual anti-angiogenic and immunomodulatory effects of HEBERSaVax (15, 16). However, its aggressive kinetics, where palpable tumors develop within days and lung metastases are detectable in 1–3 weeks, and its genetic homogeneity do not fully recapitulate the protracted timeline and heterogeneity of human melanoma (17). This rapid progression created a specific experimental constraint for our active immunization strategy. Our data demonstrate that a robust, functionally relevant anti-VEGF antibody response requires a priming period of several weeks, with significant titers and VEGF achieved only after the second and third immunizations, respectively (**Supplementary Figure S3**). Initiating vaccination after tumor implantation would therefore create a critical temporal mismatch, where the exponential tumor growth would outpace the development of protective humoral immunity. Consequently, our prophylactic-therapeutic schedule was essential to pre-establish immunity, ensuring its presence during the critical early phases of tumorigenesis to validly assess the vaccine’s mechanism of action. Importantly, this fundamental kinetic difference highlights that the time required for HEBERSaVax to induce a potent immune response is well-aligned with the more protracted progression of human melanoma, underscoring its therapeutic feasibility in a clinical setting.

Our results demonstrate that HEBERSaVax anti-VEGF antibody response significantly correlates with reduced B16-F10 tumor growth. This therapeutic effect is mechanistically supported by a marked reduction in Ki67^+^ proliferating cells, confirming the vaccine’s potent anti-proliferative activity. We propose this occurs through the effective disruption of VEGF-mediated autocrine and paracrine signaling. Previous work has demonstrated that HEBERSaVax induces patient antibodies that block VEGF binding to both VEGFR1 and VEGFR2 (4). Here, we confirm this critical functionality in our murine model, showing that vaccine-induced sera specifically inhibit the VEGF/VEGFR2 (KDR) interaction, the primary pathway mediating VEGF’s biological effects. This finding directly supports the mechanism of impaired tumor growth via blocked VEGF/VEGFR autocrine signaling. Furthermore, this same antibody response disrupts VEGF’s paracrine signaling to endothelial cells, launching a direct attack on the tumor vasculature. This is critically important, as clinical studies have firmly established that high intratumoral microvessel density (MVD) correlates with adverse prognosis in melanoma, including increased Breslow thickness and a higher risk of metastasis (10). In our study, the VEGF blockade manifested as significant reductions in both CD31^+^ microvessels and αSMA^+^ mature vessels, indicating a simultaneous attack on endothelial cells and the pericytes essential for vessel stability. The strong inverse correlation between the vaccine’s neutralizing activity and microvessel density directly links its immunogenic effect to the dismantling of the pathological vascular networks known to drive poor clinical outcomes. Future studies incorporating the evaluation of hypoxia markers (e.g., pimonidazole) (18) will be crucial to further elucidate this immune-vascular crosstalk.

Beyond angiogenesis suppression, HEBERSaVax treatment was associated with a significant increase in the infiltration of CD4^+^ and CD8^+^ T cells, a change consistent with immune-mediated tumor control. The inverse correlation between CD4^+^ T cells and microvessel density supports the concept that vascular normalization facilitates immune cell trafficking (19). Furthermore, these findings are consistent with and potentially extend studies showing that anti-VEGF therapies improve T-cell infiltration by reducing immunosuppressive factors like MDSCs and Tregs (20, 21). Collectively, the observed changes in the TME are consistent with the established immunomodulatory effects of VEGF blockade and appear to work in concert with the direct antiangiogenic effects to create a multifaceted antitumor response.

Analysis of Treg infiltration suggested a potential increase in the CD8^+^/FOXP3^+^ ratio following HEBERSaVax treatment; however, this difference did not reach statistical significance. This was likely due to the limited sample size combined with the characteristically low baseline frequency of FOXP3^+^ cells in the B16-F10 model (<5% of CD4^+^ population), which constrained the statistical power for this specific analysis. Thus, while these preliminary trends in Treg modulation are biologically encouraging and consistent with the proposed mechanism of VEGF blockade, they warrant cautious interpretation. In contrast, the significant increases in CD4^+^ and CD8^+^ T-cell infiltration, clearly captured and quantified using our spatially resolved immunohistochemical approach, represent robust evidence of the vaccine’s ability to promote a more immunologically active TME. To definitively elucidate the effects on regulatory subsets and functional immune states, future studies will employ high-dimensional immunophenotyping via multiparameter flow cytometry. This will provide more accurate absolute quantification of immune subsets, including Tregs (CD4^+^CD25^+^FOXP3^+^), and enable simultaneous evaluation of T-cell activation (e.g., CD69, CD137), exhaustion (e.g., PD-1, TIM-3, LAG-3), cytokine profiles, and the abundance of MDSCs and dendritic cell subsets. Integrating these precise functional and quantitative data with the spatial context provided by IHC will be crucial to fully decode the extent to which VEGF-specific immunity counteracts the immunosuppressive tumor milieu and to identify candidate biomarkers of treatment response.

The observed immune and vascular changes induced by HEBERSaVax align with the known goals of anti-angiogenic therapy, currently achieved with agents like bevacizumab. As the most clinically advanced agent in this class, this humanized monoclonal VEGF antibody, has demonstrated multifaceted anti-tumor mechanisms in melanoma (22). Bevacizumab´s primary mode of action involves VEGF sequestration, preventing receptor binding and subsequent pathway activation. Preclinical studies reveal three complementary effects in the vasculature: i) angiogenesis inhibition by blocking endothelial cell recruitment, ii) regression of immature tumor vasculature, and iii) structural normalization of remaining vessels (23, 24). Significantly, these vascular modifications not only directly suppress tumor growth but also enhance therapeutic efficacy, improving chemotherapeutic drug delivery while synergizing with immunotherapies through enhanced T-cell infiltration (19, 20).

While a direct preclinical comparison to species-specific VEGF inhibitors like bevacizumab is not feasible, the distinct mechanisms of action of active versus passive immunotherapy allow us to postulate key potential differentiators. Unlike the transient VEGF blockade achieved with bevacizumab, which requires frequent infusions and is associated with class-specific toxicities (22), HEBERSaVax induces an endogenous, sustained immune response. Clinical data from phase 1 and 2 trials demonstrate that vaccination can induce anti-VEGF antibody titers that persist for years with occasional boosters, suggesting a potential durability and long-term, metronomic control of VEGF (1, 6). Furthermore, while bevacizumab is associated with class-specific toxicities (25), HEBERSaVax has exhibited an exemplary safety profile across clinical trials, with no serious adverse events or classic ‘antiangiogenic-like toxicities’ reported. This is hypothetically due to the immune system’s self-regulating nature, which may avoid complete VEGF ablation and maintain physiological levels necessary for vascular homeostasis. This potential durability and a favorable safety profile could position HEBERSaVax as a promising foundational component for combination therapies. Future clinical trials designed to directly compare HEBERSaVax with standard anti-angiogenic therapies will be essential to validate these potential benefits.

Metastatic complications account for the majority of cancer-related mortality, with compelling evidence implicating angiogenesis as a critical driver of this process. The B16-F10 melanoma model has become a gold standard in metastasis research due to its aggressive, reproducible dissemination patterns and experimental versatility. Its capacity for quantitative tracking via multiple implantation routes, particularly intravenous injection for lung metastasis studies, has enabled rigorous evaluation of therapeutic strategies (26). HEBERSaVax demonstrates systemic efficacy in this context, significantly reducing pulmonary metastatic burden in the B16-F10 model. This finding holds particular clinical relevance given the lungs’ susceptibility to metastatic colonization (27) and confirms the vaccine’s ability to inhibit hematogenous dissemination, a critical benchmark for anti-metastatic therapies. This robust anti-metastatic effect is supported by the vaccine’s capacity to induce a sustained immune response (5). This sustained effect may offer a key advantage for the long-term control of metastatic disease, where chronic VEGF suppression is required. Furthermore, its favorable safety profile observed to date hypothetically enables extended treatment durations without cumulative toxicity, though direct comparative clinical trials are necessary to validate these potential benefits.

A key consideration of this study is its prophylactic-therapeutic design, necessary to evaluate mechanism in an aggressive model. While this robustly demonstrates HEBERSaVax’s capacity to induce anti-angiogenic and immunomodulatory effects, targeting a single pathway may be insufficient for advanced disease. Therefore, we envision HEBERSaVax primarily as a foundational component of combination regimens. Its ability to normalize the vascular and immune landscape positions it to synergize with immune checkpoint inhibitors (e.g., anti-PD-1). Future work must evaluate HEBERSaVax in strictly therapeutic settings and explore combination strategies, including adoptive transfer of vaccine-primed immunity, to overcome the temporal constraints of active immunization in advanced disease and fully define its clinical role.

In conclusion, our results demonstrate that HEBERSaVax effectively controls melanoma progression by simultaneously disrupting tumor angiogenesis and fostering changes in the immune microenvironment, including increased T-cell infiltration, that are consistent with a more pro-inflammatory state. These preclinical findings provide a strong rationale for its further development not as a standalone agent, but as a foundational component of combination therapies aimed at overcoming the immunosuppressive barriers of advanced melanoma.

## Data Availability

The original contributions presented in the study are included in the article/**Supplementary Material**. Further inquiries can be directed to the corresponding authors.
