# Supplementary material for: Anti-VEGF immunotherapy with HEBERSaVax suppresses melanoma growth and metastasis via angiogenesis blockade and enhanced T-cell infiltration
Source: Front Immunol. 2025 Dec 19;16:1667651. doi: 10.3389/fimmu.2025.1667651 (PMC12757277; doi:10.3389/fimmu.2025.1667651)
Supplement: Supplementary file 1 [file DataSheet1.pdf]

Supplementary Material

1 Supplementary Tables

Table S1. Chemicals and materials

1.1 Antibodies.

Primary antibodies	Species	Clonality	Dilution	Company	Reference
Alpha smooth Muscle Actin (a-SMA)	Mouse	monoclonal	1:10.000	SIGMA	a2547
CD31	Rabbit	multiclonal	1:3.000	Abcam	ab281583
Iba1	Rabbit	polyclonal	1:1.500	FUJIFILM Wako	019-19741
Ki67	Rabbit	monoclonal	Ready to use	Master Diagnostica	MAD-000310QD-3
CD4	Rabbit	multiclonal	1:1.000	Abcam	ab288724
CD8	Rabbit	monoclonal	1:2.000	Abcam	ab217344
FOXP3	Mouse	monoclonal	1:100	Thermo Fisher	14-5773-82

1.2 Secondary reagents.

Secondary reagents	Specificity	Dilution	Company	Reference
Novolink Polymer	anti-rabbit IgG	Ready to use	Leica Biosystems	RE7200-CE
ImmPRESS Polymer	anti-mouse IgG	Ready to use	Vector Laboratories	MP-7402

1.3 ELISA reactive.

Name	Supplier	Catalogue
GST-VEGF121	CIGB, Havana	-
Human VEGF R2/KDR Biotinylated Antibody	R&D Systems	BAF357
Skim milk powder	AppliChem	A0830
Tween 20	AppliChem	A1389
VEGF Receptor-2 (Flk-1, KDR)/Fc Chimera human	Sigma	V6758
Streptavidin−Peroxidase	Sigma	S5512

2 Supplementary Figures

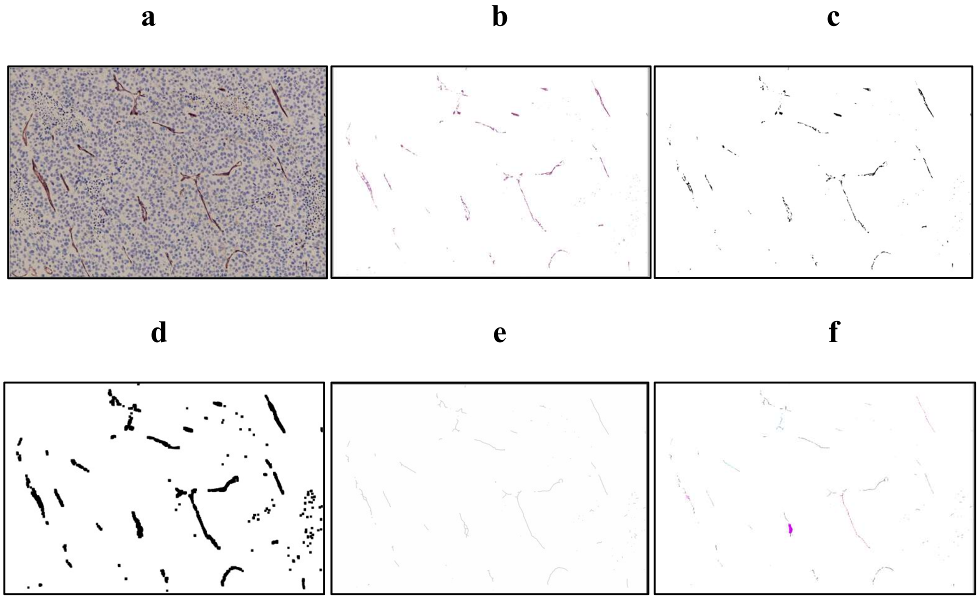

Supplementary Figure 1. Sequential steps were carried out to quantify the number of vessels per image. The original BJP image **(A)**. The BJP-staining image. inverted 8-bit gray-level digital image **(C)**. The dilated image **(D)**. Skeletonized image **(E)**. The number of vessels counted in this image (f). In this image, the number of vessels per area was 15.

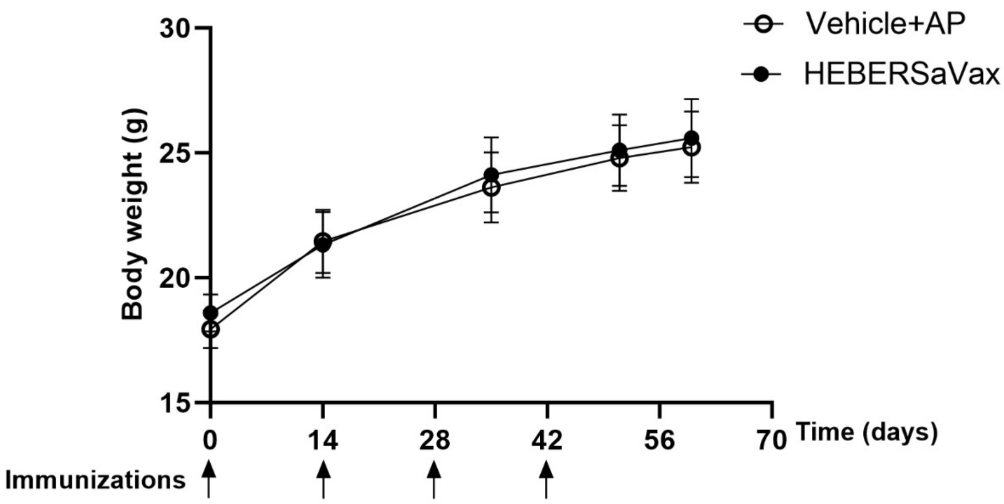

Supplementary Figure 2. Time course of body weight in mice immunized with HEBERSaVax. Each symbol represents the average and standard deviation of the body weight of the mice (n=8/group) according to the day of the immunization schedule. Arrows indicate immunization days. No differences were detected between each immunization group ($p>0.05$, Two-way ANOVA, Sidak´s post-test).

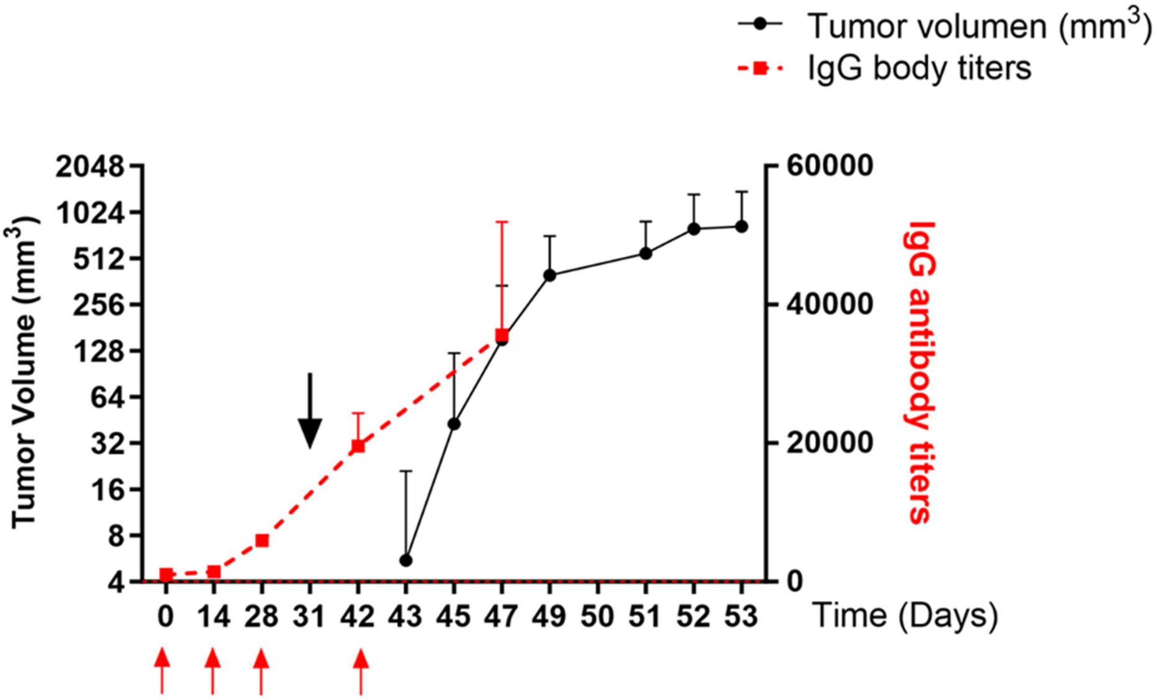

Supplementary Figure 3: Temporal association of the anti-VEGF humoral response with tumor volume in mice vaccinated with HEBERSAVAX. The kinetics of serum anti-VEGF IgG antibodies were superimposed on tumor volume curves in vaccinated mice. Three immunizations were administered prior to the tumor challenge (day 31), followed by one booster dose. A significant anamnestic antibody response was observed in sera extracted 14 days after the second immunization (p=0.0016, ANOVA, Dunn's test, Day 0 vs. Day 41), which was associated with effective tumor growth control.
